# Survival analysis and associated factors of high-grade glioma patients

**DOI:** 10.7705/biomedica.6742

**Published:** 2024-05-30

**Authors:** Lina Marcela Barrera, León Darío Ortiz, Hugo de Jesús Grisales, Mauricio Camargo

**Affiliations:** 1 Grupo de Investigación en Ciencias Médicas, Escuela Ciencias de la Vida, Programa de Medicina, Universidad EIA, Medellín, Colombia Grupo de Investigación en Ciencias Médicas Escuela Ciencias de la Vida, Programa de Medicina Universidad EIA Medellín Colombia; 2 Instituto de Cancerología, Clínica Las Américas, Medellín, Colombia Clínica Las Américas Medellín Colombia; 3 Grupo de Investigación Demografía y Salud, Facultad Nacional de Salud Pública, Universidad de Antioquia, Medellín, Colombia Universidad de Antioquia Grupo de Investigación Demografía y Salud Facultad Nacional de Salud Pública Universidad de Antioquia Medellín Colombia; 4 Dirección Científica, GenéticaLab, Medellín, Colombia GenéticaLab Medellín Colombia

**Keywords:** Glioma, risk factors, genetics, prognosis, survivorship, Kaplan-Meier estimate, glioma, factores de riesgo, genética, pronóstico, supervivencia, estimación de Kaplan-Meier

## Abstract

**Introduction.:**

High-grade gliomas are the most common primary brain tumors in adults, and they usually have a quick fatal course. Average survival is 18 months, mainly, because of tumor resistance to Stupp protocol.

**Objective.:**

To determine high-grade glioma patient survival and the effect of persuasion variables on survival.

**Materials and methods.:**

We conducted a longitudinal descriptive study in which 80 untreated recently diagnosed high-grade glioma patients participated. A survey was conducted regarding their exposure to some risk factors, degree of genetic instability in peripheral blood using micronucleus quantification on binuclear lymphocytes, micronuclei in reticulocytes and sister-chromatid exchanges in lymphocytes. In the statistical analysis, this study constructed life tables, used the Kaplan-Meier, and the log-rank test, and in the multivariate analysis, a Cox proportional hazards model was constructed.

**Results.:**

Eighty patients' clinical, demographic and lifestyle characteristics were analyzed, as well as their survival rates and the average survival time is 784 days (interquartile range: 928). Factors like age, exposure at work to polycyclic hydrocarbons and the number of sister-chromatid exchanges in lymphocytes in the first sampling was significantly survival-related in the multivariate analysis.

**Conclusion.:**

We determined that only three of the analyzed variables have an important effect on survival time when it comes to high-grade glioma patients.

## RESUMEN

**Introducción.:**

Los gliomas de alto grado son los tumores cerebrales primarios más comunes en adultos y, por lo general, tienen un curso mortal rápido. La supervivencia media es de 18 meses, principalmente, como consecuencia de la resistencia del tumor al protocolo Stupp.

**Objetivo.:**

Determinar la supervivencia de los pacientes con glioma de alto grado y el efecto de las variables de persuasión en la supervivencia.

**Materiales y métodos.:**

Se llevó a cabo un estudio descriptivo longitudinal en el que participaron 80 pacientes con diagnóstico reciente de glioma de alto grado no tratados. Se hizo una encuesta sobre su exposición a algunos factores de riesgo, grado de inestabilidad genética en sangre periférica mediante cuantificación de micronúcleos en linfocitos binucleares, micronúcleos en reticulocitos e intercambios de cromátidas hermanas en linfocitos. En el análisis estadístico, se construyeron tablas de vida, se utilizó Kaplan-Meier y la prueba de rangos logarítmicos, y en el análisis multivariado, se construyó un modelo de riesgos proporcionales de Cox.

**Resultados.:**

Se analizaron las características clínicas, demográficas y de estilo de vida de 80 pacientes, así como sus tasas de supervivencia y el tiempo medio de supervivencia fue de 784 días (rango intercuartílico: 928). Factores como la edad, la exposición laboral a hidrocarburos policíclicos y el número de intercambios de cromátidas hermanas en linfocitos en el primer muestreo se relacionaron significativamente con la supervivencia en el análisis multivariante.

**Conclusión.:**

Según los resultados, el estudio determinó que solo tres de las variables analizadas tienen un efecto importante en el tiempo de supervivencia cuando se trata de pacientes con glioma de alto grado.

Cancer is the second cause of death in the world, which represented 8.8 million deaths in 2015; it is estimated that for 2030, this figure will increase 50-60% ^1^^-^^3^. The strangest and most devastating malignancies are brain and central nervous system malignant tumors, which include more than 50 complex diseases which are diversified depending on their location, morphology, molecular biology, and clinical behavior ^4^.

This type of pathology has a worldwide 13/100.000 inhabitants/year incidence rate ^5^^,^^6^. Average survival is just 18 months, mainly, because of resistance to the most widely used therapy protocol in the world ^7^^-^^9^, which involves surgery, radiotherapy and adjuvant therapy using temozolamide which is a powerful genotoxic mutagenic alkylating agent.

Several studies have been conducted worldwide trying to elucidate which could be the risk factors associated to the development of this pathology. Up until now, it is known that high-grade gliomas are most frequently found in males, because they have been related to preponderantly male occupations as being exposed to petro-agro-chemicals, radon gas and electromagnetic waves ^3^^,^^10^. Some authors highlight the fact that the incidence rate of this pathology is increasing in a sector of a population exposed to the use of state-of-the-art technology ^5^^,^^6^^,^^11^^-^^13^. Other risk factors include being exposed to pesticides and X-rays at work, as well as family background and socioeconomic stratum ^5^^,^^14^^-^^22^.

Some studies relate the risk of having this disease with the intake of alcohol, caffeine, antihistamines, and anti-inflammatory non-steroids (AINE) ^14^^-^^22^. Nevertheless, there are other factors named protectors, which may reduce the probability of contracting this disease, like exercise and diet (eating vegetables and antioxidants).

The objective proposed for this study was to determine high-grade glioma patient survival and the effect of persuasion variables on survival. This report represents the largest study on high-grade glioma patients in just one institution in Colombia with a total of 80 patients included during a 22-month period.

## Materials and methods

### Type of study

This is a longitudinal descriptive study, in which, researchers we analyzed the possible association between high-grade glioma patient survival and variables including clinical and genetic factors, demographics, family background and lifestyles.

### Study population

The inclusion of the participants of this study was done by means of a sampling by including 80 patients with a recent diagnosis of malignant gliomas (high grade), undergoing surgical resection and attending the *Instituto de Cancerología* of the *Clínica Las Américas* in the city of Medellín, over a period of 22 months (2013-2015).

### Data gathering

Before an informed consent was approved by the Ethics Committee independent of the *Instituto de Cancerología* of the *Clínica Las Américas* and the bioethics committee at the research headquarters at *Sede de Investigación* of the *Universidad de Antioquia,* researchers used a format established to store each patient's information. It was based on a structured interview asking about demographic characteristics, personal and family background, exposure to some risk factors as a family history of cancer, lifestyle and exposure at work, and para-clinical information and the development of the disease.

An additional format was used to record conventional histopathologic results and genotoxicity tests. The histopathologic diagnostic was re-evaluated independently by two experienced neuropathologists, following the 2007 WHO classification criteria ^1^, with a 61.25% agreement percentage. Survival data were collected when patients visited the hospital during chemotherapy or via bi-monthly telephone interviews.

### Evaluation of genomic instability levels

To evaluate patient genetic instability levels, we took three peripheral blood samples (6-8 ml) in a Vacutainer-type sterilized tube with a green cap (heparinized); the first sample was taken before starting treatment (sample 1), another after 45 days of post- chemotherapy and radiotherapy (sample 2), and the last one after the first adjuvant cycle with temozolamide (sample 3). Measurements were taken using three techniques:

1. micronuclei in reticulocytes by flow cytometry following the protocol of other studies (23-25), with their modifications, in which we analyzed at least 80,000 events for each sample, and which was standardized in our Laboratory with the help of the institution's cytometry unit;
2. micronuclei in lymphocytes in 2 x 1000 cells via a conventional technique ^26^^,^^27^, and
3. sister chromatid exchange via and adapted conventional method and IPCS recommendations (2000) where 2 x 25 metafases-M2 were analyzed ^28^.

The techniques used an approach related to their sensitivity and genotoxic damage type. They detected:

1. micronuclei in reticulocytes because of the high number of events which enable the analysis of flow cytometry;
2. micronuclei in lymphocytes to quantify, principally, clastogenic events recently associated to the double chain breaks produced by radiation or by the effect of recent clastogenic events associated to double chain breaks resulting from radiation, or by the effect of post replicative adducts, and
3. sister chromatid exchange to quantify events related to a post replicative repair that can generate the adducts by alkylation like those produced by temozolamide therapeutic alkylating.

### Statistical analyses

In accordance with literature, a 540-day cutoff point was chosen as glioma patients' maximum survival time, and in accordance with this value, survival tables were constructed for the time to the event according to each of the demographic variables, clinical background, lifestyles, and genetic variables.

An assessment of the statistical significance of the total survival time regarding independent variables was conducted using the log-rank test or Tarone-Ware test.

An explicative multivariate model of Cox proportional hazards was constructed ^29^^,^^30^ following the standard methodology for that purpose. The outcome variable was glioma patients' survival time in months and the condition of no censorship referred to the fact that patients had developed the event, death. Censored subjects' information, that is, those people who did not experience the event during observation time contributed usefully to estimate the model.

The maximum likelihood method was used to estimate coefficients based on a partial likelihood function. Before constructing the multivariate model, each one of the univariate or simple Cox proportional-hazard models were analyzed. In the models, we observed each variable's levels of significance with an outcome, an HR (hazard rate), its 95% confidence interval and the Akaike information criterion point value ^31^, to have objective strategies to construct a multivariate model.

The variables which are candidates of joining the multivariate model were chosen using the Hosmer Lemeshow's criterion, that is, those that when associated with the outcome, they would have a significance level below 0.25.

After selection, the first variable entered to constitute the multivariate model was the one that had the lowest Akaike information criterion point value, and then, the other variables were incorporated in each step, in order of Akaike information criterion point value magnitude and clinical importance until the variables that made up that model would all be significant and have a lower Akaike information criterion point value.

To compare the HR change in the construction of simple models in reference to the multiple models, crude HR and adjusted HR were calculated to assess the change. The compliance of proportional risk was assessed using the statistical significance test that avails the compliance own risk when the significance of the test surpasses.

## Results

### Population general characteristics

A total of 110 patients were included in this study. At first, there was a categorization of epidemiological, clinical and research variables which were taken into account to analyze patients' information; 93 variables were collected per patient, and those variables included overall information, a history of cancer in the family, lifestyle, medication use, exposure at work, survival rates and genetic instability (in Spanish, micronuclei in lymphocytes, micronuclei in reticulocytes, sister chromatid exchange respectively).

The study populations' most important demographic characteristics are presented in table 1. Regarding age, the mean was 48 ± 24.5 years of age (interquartile range) with a mean of 49 ± 15, while, in controls the mean was 43 ± 12.7, both age groups with a normal distribution. Following the age classification established by the World Health Organization (WHO), we found that 40 of the participants (50%) were included in the 40-64-year-old range, 24 (30%) were young adults (17-37) and 16 (20%) were in the senior citizen group (> 64). In the control group, 40% were young adults, 46,6% adults and only 13,3% were older adults. Likewise, it was determined that 42 (52.5%) patients were men without statistical differences regarding women's distribution (32). Very similar numbers were found in the control group. Controls were matched by age and sex (table 1).

**Table 1:** Summary indicators of demographic variables

	Patients		Controls	
	p	Mean (SD)	p	Mean (SD)
Age in completed years	0.123*	49,0 (15.0)	0.515*	43.6 (12.7)
	n	%	n	%
Sex				
Male	42	52.5	16	53.3
Female	38	47.5	14	46.6
Age groups (years)				
Young adult (18-39)	24	30	12	40
Adult (40-64)	40	50	14	46.6
Senior citizen (≥ 64)	16	20	4	13.3
Race				
Caucasian	22	27.5	14	46.6
Amerindian	58	72.5	16	53.3
Occupation				
Farmer	15	18.8	0	0
Homemaker	10	12.5	1	3.33
Customer service and sales	17	21.3	8	26.6
Mechanics, electronics and construction	10	12.5	0	0
Chemicals and textiles	9	11.3	1	3.33
Others	19	23.8	15	50
Socioeconomic level				
Low	16	20.3	2	6.8
Medium	52	65.8	14	46.6
High	11	13.9	14	46.6

SD: Standard deviation

* Shapiro Wilk normality test

According to the patients' phenotypic traits, it was observed that 72.5% were Amerindians, and 27.5% were Caucasian. Regarding occupation, 18.8% were farmers, 21.3% worked in customer service and sales related jobs, 12.5% were homemakers, 12.5% worked in mechanics, electronics and construction, 11.3% in textiles and 23.8% in other jobs. When the socioeconomic level was analyzed, 65.8% were in a medium socioeconomic level, while 20.3% were in a low socioeconomic level (strata 1 and 2), and just 13.9% were in a high socioeconomic level.

### Description of clinical features

Three criteria were considered: tumor classification by type and histological grade and body mass index.

The tumor classification of the patients was performed in two stages; in the first stage, the pathologist provided the information based on the results of the initial pathology, with the consent of the study participants, in which 9 of 80 patients did not have an initial diagnosis (unclassified brain neoplasm) and three of them the type and grade of glioma were not determined (high grade neoplasm of glial origin).

**Figure 1:**
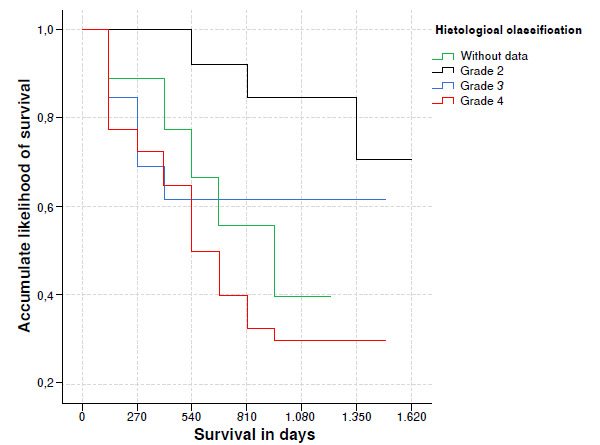
Accumulated likelihood for glioma patient survival in accordance with histological classification

Figure 1 shows the classification by type and initial histological grade. Of the 80 patients, 39 were classified as glioblastoma, 5 as oligodendroglioma-III, 2 as oligoastrocytoma-III, 2 as astrocytoma-II, 9 as astrocytoma-III, 5 as oligoastrocytomas-II, 8 as astrocytoma-II and 12 that were not classified or determined to be high grade glioma in classification.

When grouping them by histological grade according to the WHO ^33^, was founded that 39 belongs to IV grade, 16 to III grade, 13 to II grade, 12 cases were not classified.

In the second stage, the reclassification of each of the cases was carried out with the help of a neuropathology expert, where the following criteria were considered according to the WHO: histological type (astrocyte, oligodendrocytes, mixed), tumor margins (focal or diffuse ^33^, cell and vascular proliferation, cell pleomorphism and necrosis.

According to this last classification, 50 patients presented glioblastoma, 3 oligodendrogliomas -III, 3 oligodendrogliomas -II, 4 as oligoastrocytoma -II and 7 as astrocytoma -III. Regarding histological grading, 50 patients presented glioblastoma (IV grade), 16 had grade III neoplasm (astrocytoma and anaplastic oligodendroglioma) and 14 grade II (astrocytoma and diffuse type oligodendroglioma) ^33^^,^^34^. Comparing the initial and the final classifications, a discrepancy of 42% was observed in the histological findings.

Grade III and IV glial tumors are denominated high grade gliomas and are candidates for the complementary treatment to surgery with adjuvant chemotherapy and radiotherapy ^33^^,^^35^^,^^47^. However, it is important to clarify, that, in this type of pathology, an integrated diagnosis is made, which includes the type and histological grade, imaging and the expression of some protein markers ^1^^,^^4^.

In accordance with the classification of body mass index given by the World Health Organization (WHO), 50.6% of the patients weighed normally, 39.6% were overweight, and just 10.1% were obese ^36^.

### Family history and lifestyle

Most of the patients had a family history of cancer (89%), of which 16%d had glioma background, 19% leukemia, 9% thyroid cancer, 25% breast cancer and 20% had relatives with another type of neoplasm. Thirty eight point eigh per cent of the patients had no alcohol intake, 12.5% had an intake of up to 40 g of alcohol a week and 47.5% from 41 to 1,500 g of alcohol a week. Likewise, 67.5% of the patients did not smoke cigarettes, 10% just smoked from 1 to 4 cigarettes a day (low intake), and 21.3% smoked moderately to high (between 5-60 cigarettes/day).

Regarding the use of state-of-the-art technology, it was found that 40% of the patients used a computer, 17.5% up to 2 hours a day and 22.5% from 3 to 15 hours a day. Eighty-five per cent of the patients referred to the use of a cell phone, 67.5% at least half an hour a day, 17.5% up to an hour and a half and 13.8% from 1.6 to 18 hours a day. Likewise, 68.4% of the patients had a regular intake of antioxidants and 67.1% exercised weekly.

### Genetic instability level quantification

When comparing the micronuclei in lymphocytes number depending on the moment of the sampling, the mean was higher in sample 2, in comparison with the mean for that number in sample 1 and sample 3, with statistic differences; a similar result was obtained when the sister chromatid exchange number was considered where the average of sample 2 was higher than sample 1 with significant differences. The mean of the micronuclei in reticulocytes percentage was higher in sample 2 and 1 respectively, but those differences have no statistical significance (table 2).

**Table 2:** Summary of measures of genetic instability for patients with high-grade gliomas

Genetic variables	n	Mean (SD)	Median	p^*^	p^**^
			(IQ range)		
Micronuclei in lymphocytes, number per					
1,000 cells					
Sample 1	44	12.1 (8.4)	9.0(7.8)	0.000	0.000
Sample 2		20.5 (10.1)	19.0 (13.0)	0.007	
Sample 3		15.3 (76.9)	13.5 (11.6)	0.132	
Micronuclei in reticulocytes percentage					
Sample 1	44	11.5 (15.4)	4.3 (11.9)	0.000	0.074
Sample 2		18.1 (23.3)	8.4 (18.8)	0.000	
Sampe 3		6.6 (6.8)	4.5 (4.5)	0.000	
Sister-chromatid exchanges in lymphocytes					
Sample 1	69	5.4 (0.8)	5.2(1.0)	0.895	0.000
Sample 2	33	6.9 (0.6)	7.0(1.0)	0.209	

SD: Standard deviation

* Shapiro Wilk normality test

** Friedman test

The three techniques used for this study are shown and with each of the samplings (sample I, II and III of the same patient), shot I= before treatment, shot II= after finishing concomitant RT with oral temozolomide, shot III = after the first cycle of intravenous of temozolomide,

n: Number of patients who reached three intakes

p: p value

### Survival time

This research was able to determine a 75-day survival time for 80 of the patients. At the end of the study, we found out that 54.7% of the patients had passed away (41 patients) and then 50% of the cases, they had 784 days of survival or less (somewhat more than 2 years). The survival median was determined from 195.6 to 1,372.8 days, with a 95% confidence interval. Patients' survival likelihood starting from the moment they were diagnosed after 540 days decreased 6% approximately from 540 to 810 days, and it stabilized at 3%. At the end of the monitoring, the likelihood for a patient would survive 1,550 days, which was the maximum observed survival value, was 43% starting when a patient is diagnosed (table 3).

**Table 3:** Life table for high-grade glioma patient survival

Lower	Upper	Patients	Deaths	Likelihood	Accumulated	95% CI for survival	
limit	limit	(n)	(n)	of survival	likelihood of survival		
				(%)	(%)		
0.00	135.03	75	12	84	84	73.6	90.6
135.04	270.07	63	4	94	79	67.6	86.3
270.08	405.11	59	5	92	72	60.4	80.8
405.12	540.15	54	8	85	61	49.4	71.2
540.16	675.19	46	5	89	55	42.8	65.1
675.20	810.23	41	4	90	49	36.4	58.7
810.24	945.27	36	2	94	46	31.4	53.5
945.28	1080.31	32	0	100	46	26.5	48.1
1080.32	1215.35	28	0	100	46	18.4	38.4
1215.36	1350.39	21	1	94	43	5.9	20.4
1350.40	1485.43	9	0	100	43	1.1	10.22
1485.44	1550	3	0	100	43		

* Uncensored information

#### Survival and demographic variables

When the study considered glioma patient survival in days related to demographic variables, the study found that survival was greater from the moment when patients are diagnosed up to 540 days, regarding young adults (83.3%, CI (95%: 61.5% 93.4%) with significant differences comparing adults and senior citizens (p = 0.0014). The accumulated likelihood of survival also predominated, up to 540 days regarding women, who were referred to as Amerindians and who worked in customer service and sales and were in a medium socioeconomic level. However, those differences were not significant regarding Caucasian men, different occupations and a high or low socioeconomic level (table 4).

**Table 4:** Survival time indicators according to socio-demographic variables and assessment of significance

Demographical characteristic	Categories	n	Deaths n (%)	Survival time		
				Accumulated likelihood of survival * (%)	CI 95%	p**
Stages in life	Young adult	24	9 (37.5)	83.3	61.5 93.4	0.0014
	Adult	35	19 (54.3)	44.8	44.8 76.5	
	Senior citizen	16	13 (81.2)	25	7.8 47.2	
Sex	Female	37	21 (56.8)	54.1	36.9 68.4	0.4598
	Male	38	20 (52.6)	68.4	51.2 80.7	
Race	Caucasian	21	13 (61.9)	47.6	10.9 66.7	0.1771
	Amerindian	54	28 (51.9)	66.8	52.4 77.5	
Occupation	Farmer	13	8 (61.5)	53.9	24.8 76.0	0.963
	Homemaker	10	4 (40.0)	60.0	25.3 82.7	
	Customer service and sales	17	10 (58.8)	70.6	43.2 86.6	
	Auto mechanics and construction	8	4 (50.0)	50.0	15.2 77.5	
	Chemicals and textiles	9	5 (55.6)	44.4	13.6 71.9	
	Others	18	10 (55.6)	72.2	45.6 87.4	
Socioeconomic level	Low	13	8 (61.5)	53.9	24.8 76.0	0.3141
	Medium	50	24 (48.0)	68.0	53.2 79.0	
	High	11	8 (72.7)	45.5	16.7 70.7	

* It indicates the likelihood of surviving from the moment when a patient is diagnosed up to the first 540 days.

** Log-rank test for total survival compared among groups

### Survival and clinical variables

Upon 540 days, the survival of glioma patients whose histological classification was grade 2, predominated, and it differed statistically in matters concerning those whose classification was rejected or who had grade 3 or 4 [91.7% (CI 95%: 77,3% 98.9%), p = 0.015] (figure 1).

Survival up to 540 days was greater for overweight patients and patients whose tumor was in the anterior part. Nevertheless, these differences were not significant in comparison with those patients who were known to be obese or whose weight was normal (p = 0.361) or those patients who had a tumor in an unknown place or the tumor was in the anterior part (p = 0.165).

**Figure 2:**
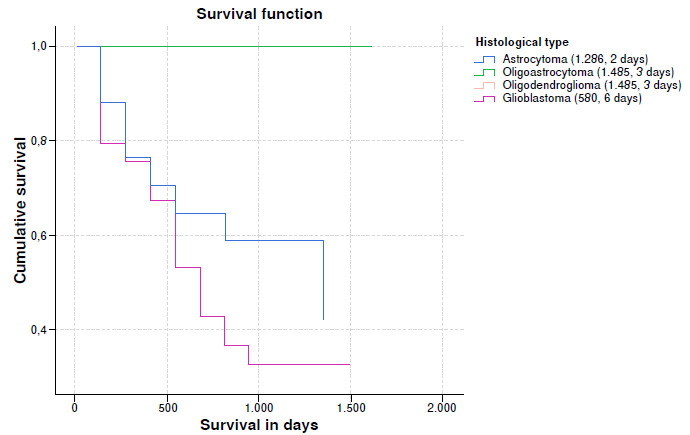
Cumulative probability for survival of patients with glioma according to histological type (n = 80 patients)

When survival rates vs. histological type were analyzed, a lower median survival was observed in glioblastomas, almost half compared to astrocytoma and one third compared to oligoastrocytoma and oligodendroglioma. An important event is that some patients with glioblastomas achieved survival rates greater than 1,000 days. It is important to clarify in the graph that the survival trend lines of oligoastrocytomas and oligodendrogliomas overlap (table 5 and figure 2).

**Table 5:** Survival indicators according to clinical variables and assessment of significance

Clinical feature	Categories	n	Deaths n (%)	Survival time		
				Cumulative probability of survival (%)	IC 85%	p**
Histological classification	Grade II	12	3 (25.0)	91.7	77.3 100.0	0.015***
	Grade III	14	5 (35.7)	64.3	43.5 95.0	
	Grade IV	49	33 (67.3)	53.1	40.8 69.0	
Tumor ubication	Without information	38	23 (60.5)	52.6	35.8 67.0	0.195***
	Front	22	9 (40.9)	72.7	49.1 85.7	
	Back	15	9 (60.0)	66.7	37.5 85.0	
BMI classification	Normal	38	21 (55.3)	52.6	35.8 67.0	0.361**
	Overweight	29	14 (48.3)	75.9	56.0 87.7	
	Obesity	7	5 (71.4)	57.1	17.283.7	

BMI: Body mass index

* Indicates the probability of surviving from diagnosis to the first 540 days

** Log rank test for overall survival compared between groups

*** Tarone-Ware test for overall survival compared between groups

Glioblastoma, astrocytomas grades II and III, oligodendrogliomas and oligoastrocytomas

### Survival and variables as background and lifestyles

Taking as reference 540 days starting when the patients were diagnosed, there were higher survival rates in patients who had no family history of cancer (leukemia, thyroid, breast) in comparison with the people that did have a family history, yet the differences were not significant.

Likewise, when aspects related to habits, customs, exposures at work, cigarette smoking, alcohol, physical exercise and using a computer were considered, there were no statistical differences in survival regarding each of the groups that form each variable.

It is worth noting that survival was more than 540 days for patients who when they were diagnosed, referred to the use of a cell phone, stated that they were exposed to aliphatic and aromatic hydrocarbons, and that they worked with hydrocarbons 2, 6 and 10 hours a day, and those who were exposed to magnetic fields a maximum of 8 hours, and had significant differences regarding the estimated survival in the other indicated variable categories (table 6).

**Table 6:** Survival time indicators in accordance with background variables lifestyles and assessment of significance

Background and lifestyles	Categories	n	Deaths n (%)	Survival time		
				Accumulated likelihood of survival* (%)	IC 85%	p**
Family history of cancer	Yes	65	36 (55.4)	61.5	48.6 72.1	0.491
	No	9	4 (44.4)	66.7	28.2 87.8	
Alcohol intake levels (g)	None	28	16 (57.1)	57.2	37.1 73.0	0.567
	From 0.1 to 40	10	7 (70.6)	60.0	25.3 82.7	
	From 41 to 1500	36	17 (47.2)	66.7	48.8 79.5	
Cigarette smoking levels (number)	None	51	25 (49.0)	66.7	52.0 77.8	0.366
	From 1 to 4	8	5 (62.5)	37.5	8.70 67.4	
	From 5 to 60	15	10 (67.6)	46.7	21.2 68.8	
Do you use a computer?	Yes	30	16 (53.3)	66.7	46.9 80.5	0.821
	No	44	24 (54.5)	59.1	43.2 71.9	
Number of hours working at a computer	None	44	24 (54.5)	59.1	43.2 71.9	0.969
	From 1 to 2	12	6 (50.0)	58.3	27.0 80.1	
	From 3 to 15	18	10 (55.6)	66.7	40.4 83.4	
Do you have a cell phone?	Yes	65	33 (50.8)	66.2	53.3 76.2	0.028
	No	9	7 (77.8)	33.3	7.8 62.3	
Time working out in hours/week	None	26	13 (50.0)	61.5	40.3 77.1	0.868
	1.0-3.0	30	18 (60.0)	56.7	37.3 72.1	
	3.1-6.0	10	5 (50.0)	70.0	32.8 89.2	
	6.1 y+	8	4 (50.0)	75.0	31.5 93.1	
Number of hours in the office for white collar workers	None	46	25 (54.3)	60.9	45.3 73.3	0.995
	From 1 to 10	28	15 (53.6)	64.3	43.8 78.9	
Number of hours a day working with hydrocarbons	None	54	34 (63.0)	57.4	43.2 69.3	0.025
	From 2.6 to 10	20	6 (30.0)	75.0	50.0 88.8	
Number of hours a day working exposed to magnetic fields	0.0-8.0	61	31 (50.8)	67.2	53.9 77.5	0.023
	9.0-16.0	13	9 (69.2)	38.5	14.1 62.8	
Number of hours a day exposed to chlorinated solvents	None	47	28(59.6)	61.7	46.3 73.9	0.327
	From 1 to 2.5	6	5 (83.3)	50.0	11.1 80.4	
	From 2.6 to 10	21	7 (33.3)	66.7	42.5 82.5	

* Indicates the likelihood of surviving from when diagnosed up to the first 540 days

** Long rank test for the total compared survival among groups

### Multivariate analysis

Aliphatic and aromatic hydrocarbon exposure, sister-chromatid exchanges in sample 1 and age what are the variables that had the most influence in the risk of dying as a result of high-grade glioma, because they met Cox proportional hazards assumption. Note that aliphatic and aromatic hydrocarbon exposure is a confusion variable: HR increased with the simple model from 2.6 to 3.2 (95% CI 1.2 - 9.0, p = 0.025), with statistical significance. It is a similar situation to what occurred with a chromatid exchange in sample 1, where there was a transition in the simple model from 1.7 to 2.3 (CI 95% 1.4 - 3.7, p = 0,001).

On the other hand, when age was taken into account for adults and senior citizens, the opposite occurred once it was controlled with sister chromatid exchange sample 1 and with age.

In accordance with the model, patients exposed to aliphatic aromatic hydrocarbons have 3.2 times the risk of dying from high-grade glioma, the whole time, adjusted using the sister chromatid exchange sample 1 and age. Similarly, said risk is 2.3 times, the whole time, in patients that had high sister chromatid exchange sample 1 level, adjusted using polycyclic hydrocarbon exposure and age. Likewise, the risk is 3.4 times (95% CI: 1.2 - 8.9, p = 0,016), the whole time, for senior citizens in comparison with young adults, adjusted using aliphatic aromatic hydrocarbon exposure and sister chromatid exchange (table 7).

**Table 7:** Variables that explain the variability regarding the risk of dying from glioma cancer.

Variables	Cox simple regression			Cox multivariate regression			
	Hazard rate	CI (95%)	p	Hazard rate	IC (95%)	p	p**
Aliphatic aromatic hydrocarbon exposure	2.6	1.1 6.2	0.031	3.2	1.2 9.0	0.025	0.8084
Chromatid exchange sample	1 1.7	1.1 2.5	0.009	2.3	1.4 3.7	0.001	0.0993
Age			0.005			0.026	0.3306
Young adult*							
Adult	1.6	0.7 3.5	0.244	1.2	0.5 2.9	0.616	0.4267
Senior citizen	4.1	1.8 9.7	0.001	3.4	1.2 8.9	0.016	0.4784

* Reference

** Cox proportional hazards assumption test

## Discussion

Glioma tumors represent most malignant brain tumors, these are low incidence neoplasms worldwide, but they are highly mortal ^6^. Nonetheless, their high relative incidence in populations of high intellectual productivity age is worrisome; in fact, gliomas are the primary brain tumors most common in adults, and unfortunately, they follow a swift fatal course. Likewise, the increase of patients in our population and among adolescents entering the use of state-of-the-art technology. It is estimated that 139,608 new cases were diagnosed in 2012 worldwide (3.9% of all cancers). It was the second most frequent cause of mortality in children and youths, and even if in the last decade, therapy has made great progress, survival increase averages are still just a few months.

In this study, we found that the median age when patients were diagnosed was 48 years, which differs from patient populations in other studies, where it surpasses 53 ^37^^-^^39^. These data are particular and differ from what has been reported in literature, in which the most affected people are youngsters and senior citizens, because according to the Colombian *Instituto Nacional de Cancerología,* central nervous system tumor incidence in Colombia is found in three age groups with the most incidence: 0-4, 15-24 and 65-79 years old ^40^.

Likewise, it was determined that the ratio of brain tumors was alike for men and women; however, it was slightly higher in men. Literature reports that a major male trend reflects observations made in other populations worldwide which has been related to higher exposure to risk factors at work ^3^^,^^20^^,^^22^.

The results observed regarding occupation agree with what was found in other studies, in which they have related the onset of that neoplasm to exposures at work to petrochemicals, agro-chemicals, pesticides, radon gas and electromagnetic waves ^3^^,^^5^^,^^6^^,^^10^^-^^13^. Most patients in the study were mid-income, and this is quite related to occupation, working conditions, income level and access to the healthcare system. Moreover, most patients belonging to this level when diagnosed worked with some exposure factors ^5^^,^^14^^-^^22^^,^^41^^-^^43^.

In clinical characteristics, the study found that most patients upon being diagnosed had a high degree of malignancy (grades 3 and 4). These results are very similar to those reported in literature, in which they observe histologically that astrocytomas evolve into high-grade gliomas, especially to glioblastomas all almost lethal and short-term ^38^^,^^44^^,^^45^. Although glioma family aggregation has been demonstrated, it is difficult to distinguish the influence of environmental exposures of hereditary background ^37^^,^^42^.

Regarding the use of state-of-the-art technology, the study found that 40% of the patients use a computer and 85% a cell phone at least half an hour a day. In 2011, the International Agency for Research on Cancer (IARC) classified the electromagnetic radio frequency in cellular phones and other devices that give off similar non-ionizing electromagnetic waves, as a "possible" human carcinogen (group 2B), as well as very low frequency magnetic fields. Furthermore, ionizing radiation is a risk factor established for brain tumors ^5^^,^^6^^,^^11^^-^^13^^,^^46^.

We report promising preliminary results that show a genomic instability increase in peripheral blood lymphocytes in a population of 80 recently diagnosed high-grade glioma patients. It is the first report of this type on gliomas, and it merits more in-depth investigation, not just because of its valuable potential, but because it is not an isolated oncological observation. In gliomas, there is a great accumulation of genetic alterations involved in the onset and progression of the disease; nonetheless, these molecular characteristics are not always a treatment response indicator. This is possibly because chemo-resistant genes are lost, or they are markers of more sensitive clones, and regarding patients' average lives, despite being biomarkers which group molecular level tumor subtypes, have very little prognostic value regarding the extension of a high-grade glioma patient's average life ^47^^,^^48^.

While measuring genetic instability, a variable range was observed regarding the inter-individual frequencies of micronuclei in lymphocytes, micronuclei in reticulocytes, and sister-chromatid exchanges in lymphocytes in patients, and this indicates a significant individual variability ^49^. The association between high genotoxicity indicator frequency and the risk of developing cancer is not confined to cancers in specific places. However, these cancer patients' frequencies reflect genetic alteration accumulation caused by endogenous and exogenous genotoxic factors, as the ones evaluated in this study, as well as individual susceptibility variations to these factors ^49^.

Patients' average survival time was 784 days (IR:928) and when conducting a comparison with different worldwide reports, we found that in all of Europe it is just 438 days for glioblastomas, 576 days in Switzerland and just 285 days for Italy. In Australia, high-grade glioma patient survival rate is 276 days, and 222 days for glioblastomas ^39^. It is evidenced that the survival rate is lower in those countries compared to Colombia. Perhaps, it is because of the prolonged use of state-of-the-art technology in those developed countries, because in the South American region the boom of these types of electronic equipment is just starting. Moreover, the type of treatment given to patients in Colombia is much more aggressive and longer than in Europe and Australia, so this could lead to a survival increase ^45^.

When conducting multivariate analyses, it was determined that just three variables have influences on high-grade glioma patient survival time: (1) age, (2) exposure to polycyclic hydrocarbons and (3) the number of glioblastomas sister chromatid exchange in sample 1 (pre-treatment). In literature, they report that these three variables may be a risk factor to get the disease. Regarding age, as it was mentioned above, high-grade glioma appearance rates increase with the passing of time because in the natural history of the disease the mutational rate may increase. Furthermore, the same mutations deteriorate cell reparation systems which leads to an accumulation of those cells altering patient survival time and a therapeutic response is added to this ^38^^,^^41^^,^^50^.

The IARC classified polycyclic hydrocarbons as possible carcinogens in 1999. The use of these chemical substances started at the beginning of the 20th century and reached its peak use from 1970 to 1980, and afterwards it reduced because of the concerns about their side effects on the environment and on public health, particularly because of their possible carcinogenicity. Nevertheless, there are no health policies which totally restrict the use of these substances, and it has been demonstrated that prolonged exposure to those substances is a risk factor for different types of cancer including leukemia, Lymphoma, kidney cancer and high-grade glioma ^51^. The amount of accumulated genetic damage caused by exposure to these substances, not only can generate the onset of the disease, but it can also alter patients' treatment response and their survival rates.

Regarding the sister chromatid exchange number in patients before treatment, it is worth noting that it is a very important piece of information because it infers the presence of high-grade glioma patient genetic instability, since chromatid sister exchanges are cytogenetic expressions of homologous recombination repair, which explains a possible accumulation of damage at a cellular level in these patients, and the development of the pathology making it more aggressive and reducing the lifetime of patients having this disease ^52^.

Finally, it is worth noting that this is the first study of this type in Colombia, in which the study makes a detailed description of the possible risk factors in the population of Colombia, and it shows that only three of the analyzed variables have an important effect on high-grade glioma patient survival time. However, further studies of this type are necessary to validate the information found in this research.

## References

[1] Louis DN, Ohgaki H, Wiestler OD, Cavenee WK, Burger PC, Jouvet A (2007). La clasificación de la OMS de 2007 de tumores del sistema nervioso central. Acta Neuropathol.

[2] WHO (2012). Media Centre. The top 10 causes of death.

[3] Piñeros M, Sierra MS, Izarzugaza MI, Forman D (2016). Epidemiología descriptiva de los cánceres de cerebro y del sistema nervioso central en Centro y Sudamérica. Cáncer Epidemiol.

[4] Louis DN, Perry A, Reifenberger G, von Deimling A, Figarella-Branger D, Cavenee WK (2016). Clasificación de tumores del sistema nervioso central de 2016 de la organización mundial de la salud: resumen. Acta Neuropathol.

[5] The International Agency for Research on Cancer (IARC) Global Cancer Observatory.

[6] Ferlay J, Soerjomataram I, Dikshit R, Eser S, Mathers C, Rebelo M (2015). Cancer incidence and mortality worldwide: Sources, methods and major patterns in GLOBOCAN 2012. Int J Cancer.

[7] Stupp R, Mason WP, van den Bent MJ, Weller M, Fisher B, Taphoorn MJ (2005). Radiotherapy plus concomitant and adjuvant temozolomide for glioblastoma. N Engl J Med.

[8] Stupp R, Hegi ME, Mason WP, van den Bent MJ, Taphoorn MJB, Janzer RC (2009). Effects of radiotherapy with concomitant and adjuvant temozolomide versus radiotherapy alone on survival in glioblastoma in a randomised phase III study: 5-year analysis of the EORTCNCIC trial. Lancet Oncol.

[9] Bleeker FE, Molenaar RJ, Leenstra S (2012). Recent advances in the molecular understanding of glioblastoma. J Neurooncol.

[10] Baldi I, Gruber A, Alioum A, Berteaud E, Lebailly P, Huchet A (2011). Descriptive epidemiology of CNS tumors in France: results from the Gironde Registry for the period 2000-2007. Neuro Oncol.

[11] Grossman SA, Ye X, Piantadosi S, Desideri S, Nabors LB, Rosenfeld M (2010). Survival of patients with newly diagnosed glioblastoma treated with radiation and temozolomide in Research studies in the United States. Clin Cancer Res.

[12] Dolecek TA, Propp JM, Stroup NE, Kruchko C (2012). CBTRUS statistical report: Primary brain and central nervous system tumors diagnosed in the United States in 2005-2009. Neuro Oncol.

[13] Alentorn A, Duran-Peña A, Pingle SC, Piccioni DE, Idbaih A, Kesari S (2015). Molecular profiling of gliomas: potential therapeutic implications. Expert Rev Anticancer Ther.

[14] Coble JB, Dosemeci M, Stewart PA, Blair A, Bowman J, Fine HA (2009). Occupational exposure to magnetic fields and the risk of brain tumors. Neuro Oncol.

[15] Michaud DS, Gallo V, Schlehofer B, Tjønneland A, Olsen A, Overvad K (2010). Coffee and tea intake and risk of brain tumors in the European Prospective Investigation into Cancer and Nutrition (EPIC) cohort study. Am J Clin Nutr.

[16] Wigertz A, Lonn S, Hall P, Feychting M (2010). Non-participant characteristics and the association between socioeconomic factors and brain tumour risk. J Epidemiol Community Health.

[17] Baglietto L, Giles GG, English DR, Karahalios A, Hopper JL, Severi G (2011). Alcohol consumption and risk of glioblastoma; evidence from the Melbourne collaborative cohort study. Int J Cancer.

[18] Cardis E, Armstrong BK, Bowman JD, Giles GG, Hours M, Krewski D (2011). Risk of brain tumours in relation to estimated RF dose from mobile phones: Results from five Interphone countries. Occup Environ Med.

[19] Claus EB, Calvocoressi L, Bondy ML, Schildkraut JM, Wiemels JL, Wrensch M (2011). Family and personal medical history and risk of meningioma. J Neurosurg.

[20] Claus EB, Calvocoressi L, Bondy ML, Schildkraut JM, Wiemels JL, Wrensch M (2012). Dental x-rays and risk of meningioma. Cancer.

[21] Scheurer ME, Amirian ES, Davlin SL, Rice T, Wrensch M, Bondy ML (2011). Effects of antihistamine and anti-inflammatory medication use on risk of specific glioma histologies. Int J Cancer.

[22] Yiin JH, Group Brain Cancer Collaborative Study, Ruder AM, Stewart PA, Waters MA, Carreón T (2012). The upper midwest health study: A case-control study of pesticide applicators and risk of glioma. Environ Health.

[23] Dertinger SD, Camphausen K, MacGregor JT, Bishop ME, Torous DK, Avlasevich S (2004). Three-color labeling method for flow cytometric measurement of cytogenetic damage in rodent and human blood. Environ Mol Mutagen.

[24] Dertinger SD, Miller RK, Brewer K, Smudzin T, Torous DK, Roberts DJ (2007). Automated human blood micronucleated reticulocyte measurements for rapid assessment of chromosomal damage. Mutat Res Genet Toxicol Environ Mutagen.

[25] Dertinger SD, Torous DK, Hayashi M, MacGregor JT (2011). Flow cytometric scoring of micronucleated erythrocytes: An efficient platform for assessing in vivo cytogenetic damage. Mutagenesis.

[26] Fenech M, Chang WP, Kirsch-Volders M, Holland N, Bonassi S, Zeiger E (2003). HUMN project: Detailed description of the scoring criteria for the cytokinesis-block micronucleus assay using isolated human lymphocyte cultures. Mutat Res Genet Toxicol Environ Mutagen.

[27] Fenech M (2007). Cytokinesis-block micronucleus cytome assay. Nat Protoc.

[28] Perry P, Wolff S (1974). New Giemsa method for the differential staining of sister chromatids. Nature.

[29] Collett D (2015). Modelling survival data in medical research.

[30] Kleinbaum D, Kupper L Nizam A, Rosenberg E (2013). Applied regression analysis and other multivariable methods.

[31] Burbine A, Fryer D, Sturtevant J, Sturtevant JL, Capodieci L (2015). Akaike information criterion to select well-fit resist models.

[32] WHO (2015). Población (OMS).

[33] Louis D, Ohgaki H, Wiestler O, Cavenee W, Burger P, Jouvet A (2007). The 2007 WHO Classification of Tumours of the Central Nervous System. Acta Neuropathol.

[34] Louis DN, Perry A, Reifenberger G, von Deimling A, Figarella-Branger D, Cavenee WK (2016). The 2016 World Health Organization Classification of Tumors of the Central Nervous System: A summary. Acta Neuropathol.

[35] Marumoto T, Saya H (2012). Molecular biology of glioma. Adv Exp Med Biol.

[36] WHO (2004). Obesidad y sobrepeso.

[37] Fisher JL, Schwartzbaum JA, Wrensch M, Wiemels JL (2007). Epidemiology of brain tumors. Neurol Clin.

[38] Crocetti E, Trama A, Stiller C, Caldarella A, Soffietti R, Jaal J (2012). Epidemiology of glial and non-glial brain tumours in Europe. Eur J Cancer.

[39] Yang P, Wang Y, Peng X, You G, Zhang W, Yan W (2013). Management and survival rates in patients with glioma in China (2004-2010): A retrospective study from a single-institution. J Neurooncol.

[40] Instituto Nacional de Cancerología (2008). Anuario Estadístico.

[41] Casartelli G, Dorcaratto A, Ravetti JL, Sola S, Vitali A, Merlo DF (2009). Survival of high grade glioma patients depends on their age at diagnosis. Cancer Biol Ther.

[42] Cabaniols C, Giorgi R, Chinot O, Ferahta N, Spinelli V, Alla P (2011). Links between private habits, psychological stress and brain cancer: a case-control pilot study in France. J Neurooncol.

[43] Plascak JJ, Fisher JL (2013). Area-based socioeconomic position and adult glioma: A hierarchical analysis of surveillance epidemiology and end results data. PLoS One.

[44] Mukerji N, Rodrigues D, Hendry G, Dunlop PRC, Warburton F, Kane PJ (2008). Treating high grade gliomas in the elderly: the end of ageism?. J Neurooncol.

[45] View of neuropsychological characteristics of patients with glioma treated in the Institute of cancer of Medellín, Colombia. Incompleto ¿Año, volume, páginas? Actaneurologica.com.

[46] Hardell L, Carlberg M, Hansson Mild K (2013). Use of mobile phones and cordless phones is associated with increased risk for glioma and acoustic neuroma. Pathophysiology.

[47] Olar A, Sulman EP (2015). Molecular markers in low-grade glioma-toward tumor reclassification. Semin Radiat Oncol.

[48] Lu J, Cowperthwaite MC, Burnett MG, Shpak M (2016). Molecular predictors of long-term survival in glioblastoma multiforme patients. PLoS One.

[49] Chang P, Li Y, Li D (2011). Micronuclei levels in peripheral blood lymphocytes as a potential biomarker for pancreatic cancer risk. Carcinogenesis.

[50] Minniti G, Enrici RM (2014). Radiation therapy for older adults with glioblastoma: Radical treatment, palliative treatment, or no treatment at all?. J Neurooncol.

[51] Neta G, Stewart PA, Rajaraman P, Hein MJ, Waters MA, Purdue MP (2012). Occupational exposure to chlorinated solvents and risks of glioma and meningioma in adults. Occup Environ Med.

[52] Lim YC, Roberts TL, Day BW, Harding A, Kozlov S, Kijas AW (2012). A role for homologous recombination and abnormal cell-cycle progression in radioresistance of glioma-initiating cells. Mol Cancer Ther.

